# ﻿Nomenclature of the veins of the fore wings of male scale insects (Hemiptera, Coccomorpha)

**DOI:** 10.3897/zookeys.1136.89528

**Published:** 2022-12-19

**Authors:** San-an Wu, Han Xu

**Affiliations:** 1 The Key Laboratory for Silviculture and Conservation of Ministry of Education, Beijing Forestry University, Beijing 100083, China Beijing Forestry University Beijing China

**Keywords:** Coccoids, forewing, new nomenclature system, Qinococcidae, *Qinococcuspodocarpus*, wing veins

## Abstract

The venation of the fore wings of male scale insects is strongly reduced and the nomenclature used for each vein is inconsistent among taxonomists. This paper reviews the different nomenclatural systems in the wing venation of male scale insects that have been suggested previously and puts forward a new system based mainly on newly found wing venation in males of *Qinococcuspodocarpus* Wu, 2022 (Hemiptera: Coccomorpha: Qinococcidae).

## ﻿Introduction

Comparative morphology of wing venation plays an important role in insect classification and phylogeny (Yang et al. 2012; Perrard et al. 2016). However, in the Coccomorpha, the nomenclature for the wing veins varies in interpretation among taxonomists, impeding phylogenetic research within the infraorder. Based on a review of the different nomenclatural systems proposed previously and a newly found wing venation pattern in males of *Qinococcuspodocarpus* Wu, 2022 (Hemiptera: Coccomorpha: Qinococcidae), a new nomenclature system for the wing venation of scale insects is suggested.

## ﻿Review of the nomenclature used for the veins of the fore wings of male scale insects

The scale insects belong to the infraorder Coccomorpha (Fallen, 1814), suborder Sternorrhyncha, order Hemiptera. They are sexually dimorphic, the adult female being wingless whilst the adult male (of winged species) has two pairs of wings; the fore wings are reasonably well developed, folding flat over the abdomen when at rest and overlapping each other, whereas the hind wings are reduced to hamulohalteres (Hodgson and Foldi 2006; Hodgson 2020).

The fore wings of male scale insects are normally large and quite broad with a narrow base and a broadly rounded apex (e.g., *Ortheziaurticae* (Linnaeus, 1758) and *Asiacornococcuskaki* (Kuwana in Kuwana and Muramatsu 1931)) (Hodgson and Foldi 2006) but are wider at the base and narrower at the apex in some species (e.g., *Drosicha* sp.). They are membranous throughout except for (usually) a longitudinal sclerotized (leather-like) thickening running parallel to and just posterior to the anterior margin called the Costal thickening (**Ct**). Compared with psyllids and aphids, the remaining wing venation in male scale insects is much more reduced, usually with two well-developed veins (or vein complexes, each composed of two veins) and some transparent but obvious lines (weakly developed veins or folds). The two main veins are the Anterior Vein (**AV**) just posterior to the Costal thickening (**Ct**), and the Posterior Vein (**PV**), which runs diagonally from near the base of the Anterior Vein (**AV**) obliquely to the margin of the Posterior Vein (**PV**); both the **AV** and the **PV** normally join at an acute angle in the basal area. Moreover, each wing also has either a long fold or a small alar lobe on its proximal hind margin, providing a structure for connecting with hooked haltere setae on the hind wing (hamulohalteres) (Hodgson 2020). The venation in the superfamily Orthezioidea (= Archaeococcoidea) is more complex than that in the superfamily Coccoidea (= Neococcoidea) (Giliomee 1961, 1967a; Hodgson and Foldi 2006).

Some workers have tried to analyze, interpret, and name the wing veins and lines, but the results have been variable and currently there is no standardized system for wing venation nomenclature. Patch (1909), after studying the homologies of the wing veins of aphids, psyllids, aleurodids, and coccoids, considered that the fore wings of *Dactyopius* sp. (Dactylopiidae) and *Planococcuscitri* (Risso, 1813) (Pseudococcidae) were composed of a short sclerotized Subcosta (**Sc**), and well-developed Radial sector (**Rs**) and Media (**M**) in accordance with the Comstock-Needham system (Comstock and Needham 1898).

Morrison (1928) considered that the venation of the fore wing of Margarodidae*sensu lato* was composed of the costal complex, the basal diagonal vein, and an apical diagonal vein, which is poorly developed, short, and occasionally present. He also believed that: 1) the costal complex contained two veins, the Subcosta (**Sc**) and Radius (**R**); 2) the basal diagonal vein, considered by Patch to be the Media (**M**) of the Comstock-Needham system, should be the Cubitus (Cu); and 3) that the apical diagonal vein was the Radial sector (**Rs**).

Ghauri (1962) considered the two wing veins of the Diaspididae to be the Radius (**R**)and Media (**M**). This was also the case for the wing veins of the Pseudococcidae (Giliomee 1961; Afifi 1968), the Coccidae (Giliomee 1967b) and the Eriococcidae (Afifi 1968).

Afifi (1968) considered that the wing veins of Pseudococcidae and Eriococcidae were comprised of the Radius (**R**)and Media (**M**) only.

**Figure 1.**
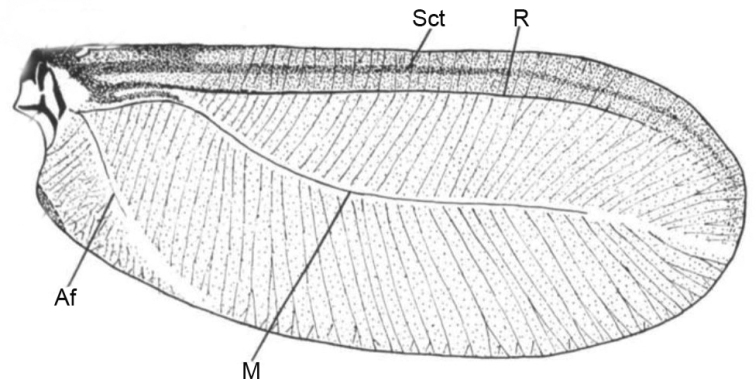
The fore wing of *Matsucoccusbisetosus* (after Beardsley 1968). Abbreviations: Af, Anal fold; M, Media; R, Radius; Sct, Subcostal thickening.

Beardsley (1968) considered that the wing venation of *Matsucoccusbisetosus* Morrison, 1939 (Matsucoccidae) was composed of a Subcostal thickening (**Sct**), a Radius (**R**), a Media (**M**), and the Anal fold (**Af**) (Fig. 1).

**Figure 2.**
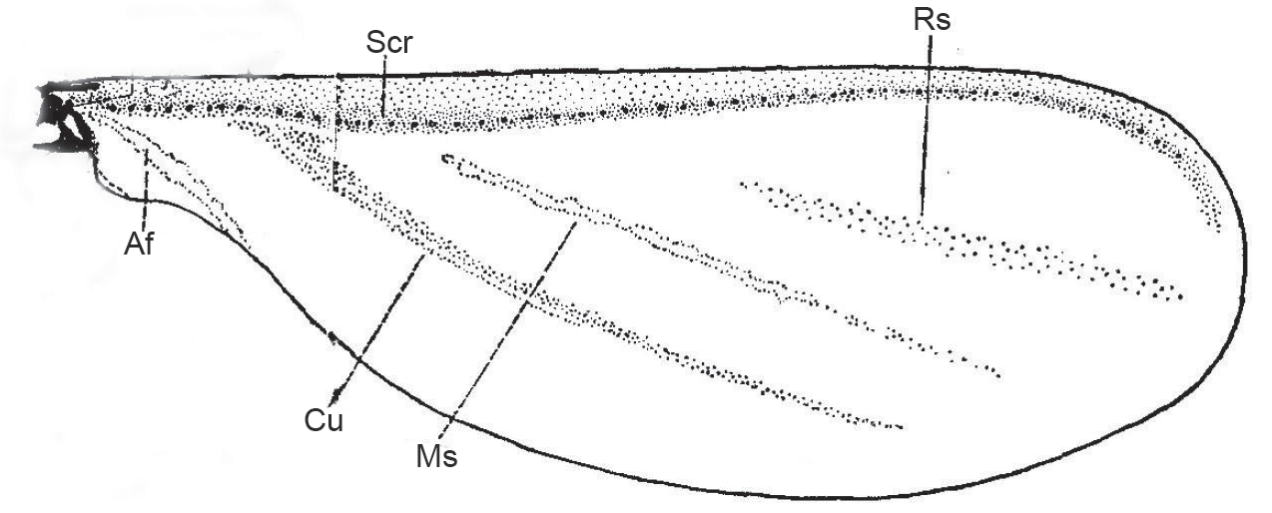
The fore wing of *Ortheziaurticae* (after Koteja 1986). Abbreviations: Af, Anal fold; Cu, Cubitus; Ms, Medial sector; Rs, Radial sector; Scr, Subcostal ridge.

Koteja (1986) considered that, on the fore wing of *Ortheziaurticae* (Ortheziidae), the Anterior Vein (**AV**) was the Subcostal ridge (**Scr**), the Posterior Vein (**PV**) was the Cubitus (**Cu**), and the two obvious lines between the AV and the PV were the Radial sector (**Rs**) and Medial sector (**Ms**), respectively, whilst the obvious line posterior to the PV was the Anal fold (**Af**) (Fig. 2).

**Figure 3.**
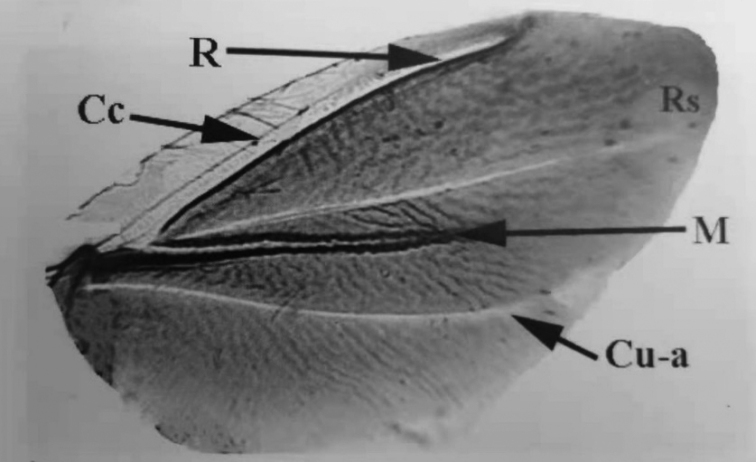
The fore wing of *Drosicha* sp. (after Lambdin 2001). Abbreviations: Cc, Costal complex (Costa + Subcosta); Cu-a, Cutino-anal vein; M, Media; R, Radius; Rs, Radial sector.

Lambdin (2001) considered that the wing venation in the more “advanced” scale insects such as *Pseudococcus* (Pseudococcidae), was composed of a Radius (**R**) and a Media (**M**), whilst the venation in wings of the more “primitive” scale insects, such as *Drosicha* sp. (Monophlebidae), consisted of a costal complex (**Costa + Subcosta**), a Radius (**R**) and a Media (**M**), whereas the white line between AV and PV was termed the Radial sector (**Rs**), and the pale line posterior to the PV was the Cutino-anal vein (**Cu-a**) (Fig. 3).

Hodgson and Foldi (2006) followed Beardsley (1968), namely identifying the fore wing of *Drosicha* to be a Media and a Subcostal thickening, the Radius.

**Figure 4.**
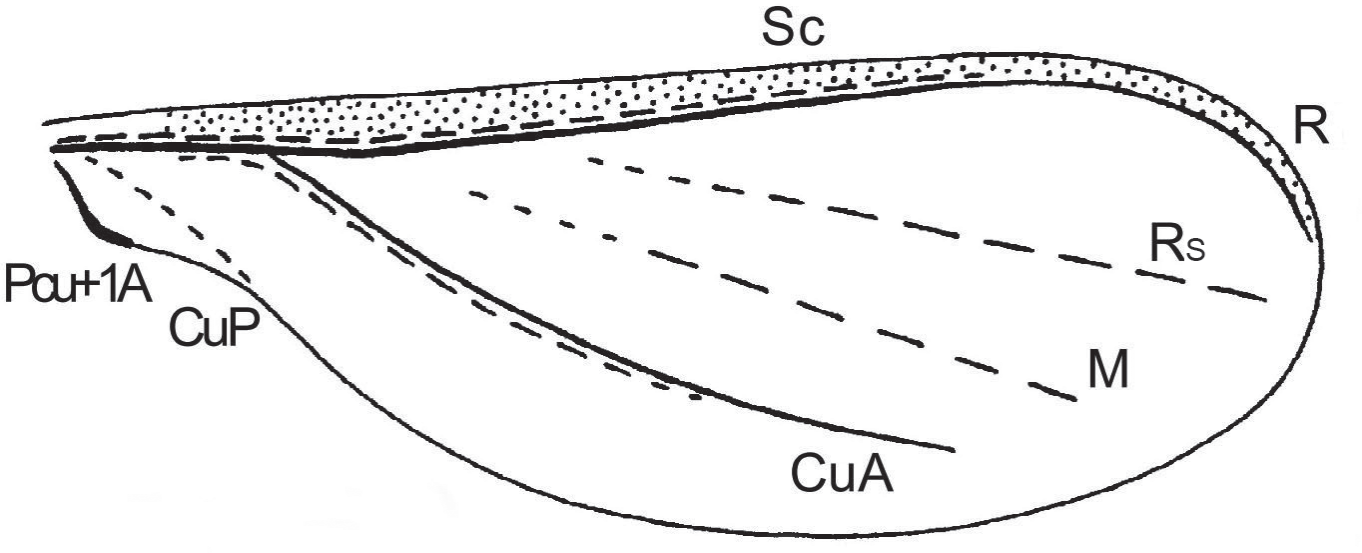
The fore wing of *Ortheziaurticae* (after Shcherbakov 2007). Abbreviations: CuA, Cubitus Anterior; CuP, Cubitus Posterior; M, Media; Pcu+1A, fused Postcubitus and anal first vein; R, Radius; Rs, Radial sector; Sc, Subcosta.

Shcherbakov (2007) considered that the venation of *Ortheziaurticae* (Ortheziidae) consisted of the Sc+R (= Costal complex of Morrison (1928)), a Cubitus Anterior (**CuA**), a Radial sector (**Rs**) (= apical diagonal vein of Morrison (1928)), whilst the pale line between the AV and the PV was the Media (**M**) and the pale line posterior to the PV was the Cubitus Posterior (**CuP**) (= Anal fold of Beardsley (1968)). Shcherbakov also considered that the anal lobe or pocket was the fused Postcubitus and anal first vein (**Pcu+1A**), and the Sc+R and the CuA were composed of two folds, each with a convex and a concave part (Fig. 4).

**Figure 5.**
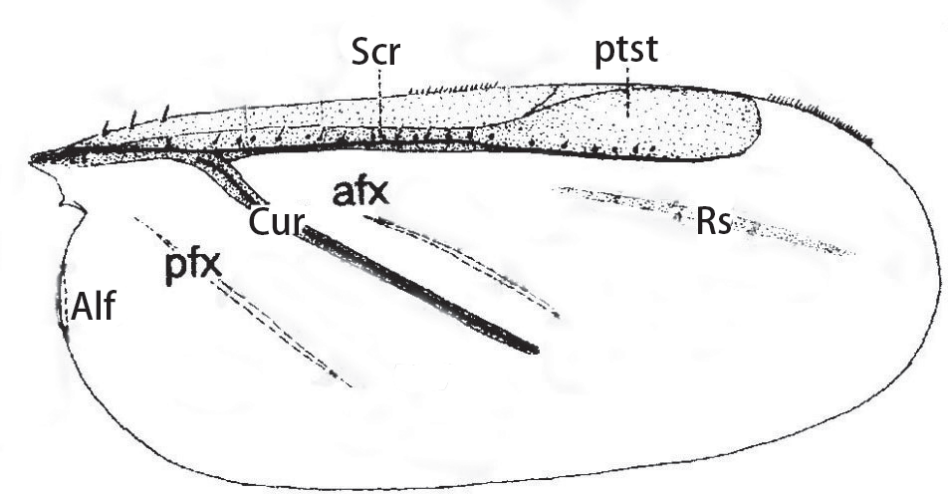
The fore wing of *Weitschatusvysniauskasi* (after Koteja 2008). Abbreviations: Alf, Alar fold; afx, anterior flexing patch; Cur, Cubital ridge; pfx, posterior flexing patch; ptst, pterostigma; Rs, Radial sector; Scr, Subcostal ridge.

Koteja (2008) explained the wing venation of the extinct species *Weitschatusvysniauskasi* Koteja, 2008 (Weitschatidae) as follows: the Subcostal ridge (Scr) runs along costal margin from the wing base toward the wing apex; the Cubital ridge (**Cur**) originates from the Scr at ~ 1/5 wing length and runs obliquely to the posterior wing margin, and the Radial sector (**Rs**) (= anterior diagonal vein of Morrison (1928)) is a slightly sclerotized oblique patch posterior to the Scr. He also described and illustrated an anterior flexing patch (**afx**) between Scr and Cur, a posterior flexing patch (**pfx**) posterior to the Cur, and a pterostigma (**ptst**) at the apex of the Scr (Fig. 5).

**Figure 6.**
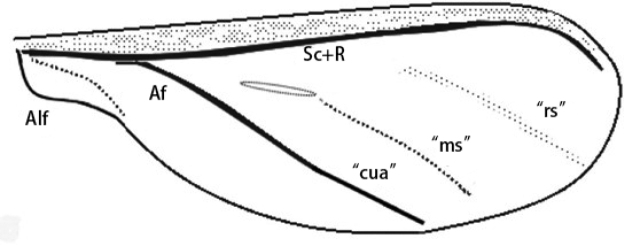
The fore wing of *Ortheziaurticae* (after Franielczyk-Pietyra et al. 2018). Abbreviations: Af, Anal fold; Alf, alar fold of anal lobe; cua in quotes, putative cubitus anterior; ms in quotes, putative media sector; rs in quotes, putative radius sector; Sc+R, fused Subcosta and Radius.

Franielczyk-Pietyra et al. (2018) explained the wing venation of *Ortheziaurticae* (Ortheziidae) as Sc+R and CuA with the two obvious lines between those two which were identified as rs (putative Rs) and ms (putative Ms), respectively (Fig. 6).

**Figure 7.**
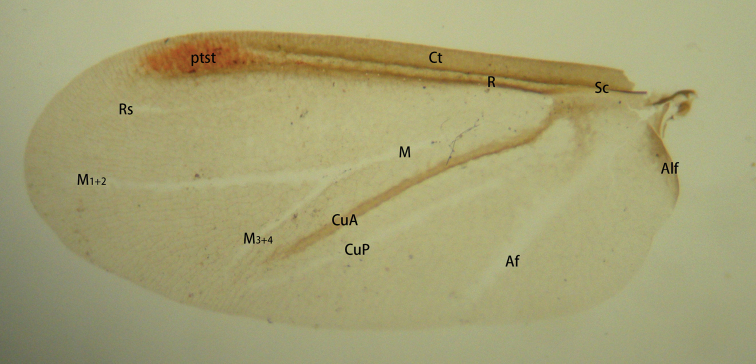
The fore wing of *Qinococcuspodocarpus* Wu. Abbreviations: Af, Anal fold; Alf, alar fold; Ct, Costal thickening; CuA, Cubitus Anterior; CuP, Cubitus Posterior; M, Media; M_1+2_, fusion of the first and second branch of media; M_3+4_, fusion of the third and fourth branch of media; ptst, pterostigma; R, Radius; Rs, Radius sector; Sc, Subcosta.

**Figure 8.**
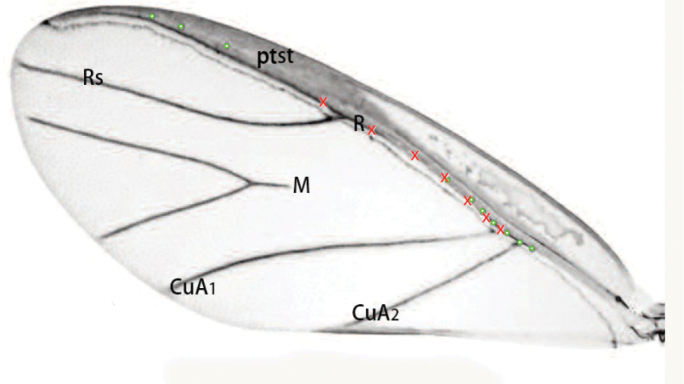
Fore wing of *Mindarus* (after Montagano and Favret 2016). Abbreviations: CuA_1_, the first branch of cubitus anterior; CuA_2_, the second branch of cubitus posterior; M, media; R, radius; Rs, radius sector. X-markers in red indicate campaniform sensilla located dorsally, O-markers in green are ventrally located sensilla.

In 2021, Wu et al. (2022) collected *Qinococcuspodocarpus* Wu (Qinococcidae), a new species in which the venation of the fore wing of the adult male is more complex than in previously described Coccomorpha. The fore wing of this species has a pterostigma, an obvious forked line between the AV and the PV (= **R** and **CuA** in Fig. 7), and two obvious lines posterior to the PV (Fig. 7). The venation of the fore wings of *Q.podocarpus* is very similar to that of *Mindarus* (Hemiptera: Aphidomorpha) (see fig. 8 of Montagano and Favret 2016, here as Fig. 8), differing mainly in that, in the former species, the veins of the Rs and M are much reduced and both an Anal fold (**Af**) and Alar fold of the anal lobe (**Alf**) are present.

## ﻿The names of the fore wing veins of scale insects

The veins of insects are composed of nerves, tracheae, and a cavity for the haemolymph (Dudley 2000) and the nomenclature used to describe them is based on their positions and relationships on each wing. However, in practice, it is impossible to name each vein based on these structures, especially for fossil examples. To understand the evolution of a target group (e.g., Coccomorpha), it is important to compare the venation of this group with that of its sister group (in this case, Aphidomorpha) and other closely related groups.

The infraorder Coccomorpha (scale insects) belongs to the suborder Sternorrhyncha which includes three other infraorders: Psyllomorpha (jumping plant-lice), Aleyrodomorpha (whiteflies), and Aphidomorpha (aphids). For the choice of names for the veins of scale insects, references to those given to the veins of aphids and the jumping plant-lice are obviously very helpful.

The Sternorrhyncha belong to the Paraneoptera within the Hemiptera. The wing venation characters of the Paraneoptera, proposed by Grimaldi and Engel (2005), are “CuA and M basally fused to the R in a common stem, and both distally emerging again from this stem, either together or separately; a cross-vein CuA-CuP present or absent, but when present, its proximal part is concave and its distal part is convex (in some taxa, the distal part can be “captured” by the CuA, so that the CuA-CuP seems to continue from the distal part of the CuA); anal area generally rather reduced, especially in forewing with two anal veins or less; CuP simple and concave; CuA convex and with two distal branches or less; M more concave that surrounding veins R and CuA, at least in its distal part, with four branches or less”. Based on this hypothesis, we have analyzed each vein on *Q.podocarpus* and have provided names for each of the veins, resulting in a new nomenclature system for the scale insects (Fig. 7) which follows.

1. Unlike in the Aphidomorpha and Psyllomorpha, the anterior margin of the fore wing in the Coccomorpha is usually less developed, with no distinct cavity; the Coccomorpha also lack tracheae, and usually have a sclerotized part posterior to the margin, so there is no costal vein present (Franielczyk-Pietyra et al. 2018); Lambdin (2001) named this structure the Costal complex. Comparing the fore wing of some jumping plant-lice (such as *Pachypsylloidescitreus* Loginova) with the anterior sclerotized part in the Coccomorpha, we consider that the best name for this structure in the Coccomorpha is the Costal thickening (**Ct**).
2. The anterior vein in the Coccomorpha runs along the costal margin from the wing base toward the wing apex, and this was named the Subcostal ridge (**Scr**) by Koteja (1986, 2008). However, Shcherbakov (2007) considered this vein to be a merged vein in *Ortheziaurticae* and named it as Sc + R (the fused Subcosta and Radius) and this was confirmed by Franielczyk-Pietyra et al. (2018). In *Matsucoccus* and *Q.podocarpus*, there are two distinct, separate veins present, and these were given the names Subcostal thickening (**Sct**) and Radius respectively by Beardsley (1968) and Hodgson and Foldi (2006). Franielczyk-Pietyra and Wegierek (2017, 2019) and Franielczyk-Pietyra et al. (2018) considered that the Sc was absent in all Sternorrhyncha but present in the Coccomorpha. However, Hong (1999) considered some aphids such as *Paroviparosiphumopimum* Zhang to have a free Sc. Comparing the venation on Coccomorpha with that of aphids, we consider the best name for (i) the anterior vein is Sc, and (ii) that, for the root stem of the posterior vein is R+M+CuA, with the top part as R, although the root stem of the posterior vein is weak in *Q.podocarpus*.
3. In the Coccomorpha, the posterior vein originates from the R+M+CuA at about 1/5 along the wing length and runs obliquely to the posterior wing margin; this is termed the basal diagonal vein by Morrison (1928), M by Lambdin (2001) and Hodgson and Foldi (2006), Cu by Koteja (1986), CuA by Shcherbakov (2007) and Franielczyk-Pietyra et al. (2018), and Cubital ridge (**Cur**) by Koteja (2008). Here we consider it to be the CuA.
4. The vein that Morrison (1928) called the anterior diagonal vein is a slightly sclerotized oblique patch (e.g., in *Ortheziaurticae* and *Xylococcuscastanopsis* Wu & Huang, 2017) or a short obvious line (e.g., in *Kuwaniabipora* Borchsenius, 1960 and *Q.podocarpus*) posterior to the radius (**R**). This vein is named the Radial sector (**Rs**) by Koteja (1986, 2008), Shcherbakov (2007), and Franielczyk-Pietyra et al. (2018). Here we believe it is best to call this vein the Rs.
5. The obvious patch or light line between R and CuA is usually unbranched and is named Ms by Koteja (1986), Rs by Lambdin (2001), and M by Beardsley (1968), Shcherbakov (2007) and Franielczyk-Pietyra et al. (2018); also, the anterior flexing patch (**afx**) by Koteja (2008). Here we consider it to be the Media (**M**). In *Q.podocarpus*, this pale line is branched, and we refer to the two branches as M1+2 and M3+4, when the pairs are present. Although Koteja (2008) considered this vein to be afx, we think this is an error because this vein has no flexing function.
6. In previous descriptions of wing venation in the Coccomorpha, there is at most one obvious line posterior to the CuA: this line has a flexing function when the wing is folded flat over the abdomen, so here we consider it to be the Anal fold (**Af**), although it was named Cubito-anal vein (**Cu-a**) by Lambdin (2001); Cubitus posterior (**CuP**) by Shcherbakov (2007), and the posterior flexing patch (**pfx**) by Koteja (2008). This fold is possibly homologous to the claval fold in Psyllomorpha.

The fore wing of *Q.podocarpus* has two obvious lines posterior to the Cubitus Anterior (**CuA**), with the posterior line having a flexing function. Here we consider the name Anal fold (**Af**) to refer to the posterior line, and the anterior line to be the Cubitus Posterior (**CuP**) in agreement with Grimaldi and Engel (2005). Comparing the male scale insect venation with that of aphids, this anterior line should be the CuA_2_. If so, the CuA is more correctly referred to as the CuA_1_.

1. Shcherbakov (2007) considered that there is a Pcu+1A vein on the anal lobe fold, but this was not confirmed by Franielczyk-Pietyra et al. (2018). Here we agree with the latter authors and consider that there is no Pcu+1A on the anal lobe fold of *Q.podocarpus*.

**Figure 9.**
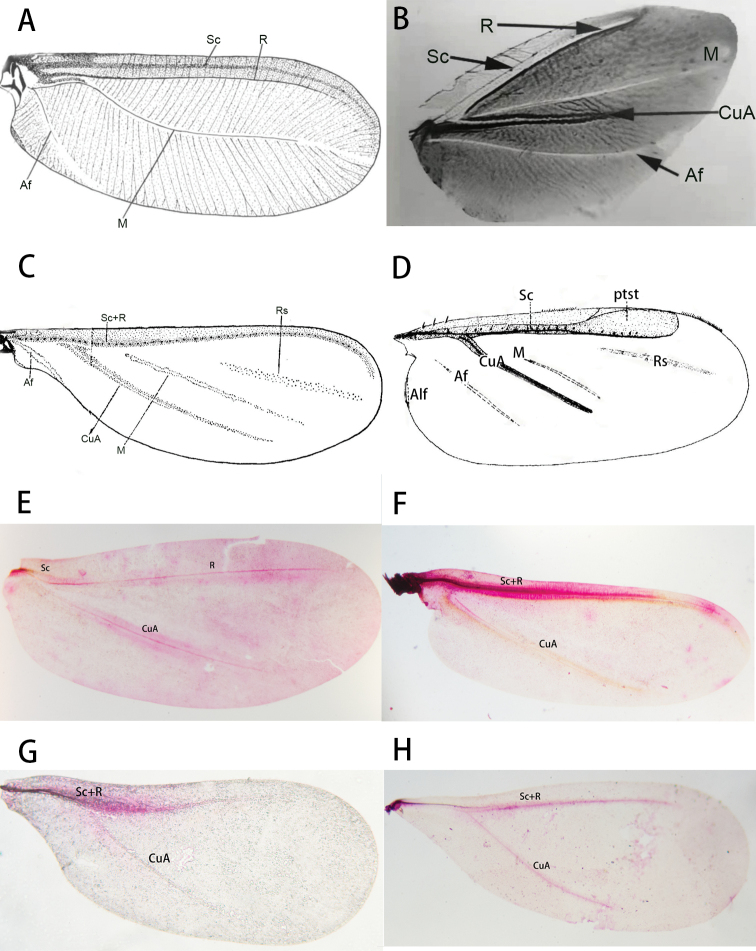
Wing venation of archaeococcoids and neococcoids **A***Matsucoccusbisetosus* (Matsucoccidae) **B***Drosicha* sp. (Monophlebidae) **C***Ortheziaurticae* (Ortheziidae) **D***Weitschatusvysniauskas* (Weitschatidae) **E***Phenacoccusfraxinus* (Pseudococcidae) **F***Parthenolecaniumcorni* (Coccidae) **G***Asiacornococcuskaki* (Eriococcidae) **H**Diaspididae sp. Abbreviations: Af, Anal fold; Alf, Alar fold; CuA, Cubitus Anterior; M, Media; ptst, pterostigma; R, Radius; Rs, Radial sector; Sc, Subcosta; Sc+R, fusion of Subcosta and Radius.

In Fig. 9, the wing veins and folds of *Matsucoccusbisetosus* in Monophlebidae, *Drosicha* sp. in Monophlebidae, *Ortheziaurticae* in Ortheziidae, *Weitschatusvysniauskasi* in Weitschatidae, *Phenacoccusfraxinus* Tang, 1977 in Pseudococcidae, *Eulecaniumtiliae* (Linnaeus, 1758) in Coccidae, *Asiacornococcuskaki* in Eriococcidae, and Diaspididae sp., are labeled based on our interpretation of the wing venation of male scale insects.

The different nomenclature used for the veins of the fore wing of adult male Coccomorpha are summarized in Table 1.

**Table 1.** Fore wing vein nomenclature for male scale insects. Abbreviations: afx: anterior flexing patch; Af: Anal fold; C: Costa; Cc: Costal complex (Costa + Subcosta); Ct: Costal thickening; Cu: Cubitus; Cur: Cubital ridge; CuA: Cubitus Anterior; Cu-a: Cubito-anal vein; CuP: Cubitus Posterior; M: Media; Ms: Medial sector; Pcu+1A: fused Postcubitus and anal first vein; pfx: posterior flexing patch; R: Radius; Rs: Radial sector; Sc: Subcosta; Scr: Subcostal ridge; Sct: Subcostal thickening.

Author	Genus	veins and/or lines										
This study	*Qinococcus*	Ct		Sc	R	Rs	M_1+2_	M_3+4_	CuA	CuP	Af	
Koteja (2008)	*Weitschatus*	–		Scr		Rs	afx		Cur	–	pfx	
Franielczyk-Pietyra et al. (2018)	*Orthezia*	–		Sc+R		Rs	‘m’		CuA	–	Af	
Shcherbakov (2007)	*Orthezia*	–		Sc+R		Rs	M		CuA	–	CuP	Pcu+1A
Koteja (1986)	*Orthezia*	–		Scr		Rs	Ms		Cu	–	Af	
Lambdin (2001)	*Drosicha*	Cc			R	–	Rs		M	–	Cu-a	
Beardsley (1968)	*Matsucoccus*	–	Sct		R	–	M			–	Af	

**Note**: some authors use capital letters and others use small caps or a combination of capital letters and small caps for the abbreviation of wing veins and folds; however, these are written in the above Table according to the abbreviations given in this section to avoid confusion.
